# A Computational Study on Thiourea Analogs as Potent MK-2 Inhibitors

**DOI:** 10.3390/ijms13067057

**Published:** 2012-06-08

**Authors:** Ming Hao, Hong Ren, Fang Luo, Shuwei Zhang, Jieshan Qiu, Mingjuan Ji, Hongzong Si, Guohui Li

**Affiliations:** 1 Department of Materials Science and Chemical Engineering, Dalian University of Technology, Dalian 116023, China; E-Mails: dluthm@yeah.net (M.H.); zswei@dlut.edu.cn (S.Z.); 2 Department of Ophthalmology, Qi Lu Hospital, Medical School of Shandong University, Jinan 250012, China; E-Mail: renhong999@sina.com; 3 Laboratory of Molecular Modeling and Design, State Key Laboratory of Molecular Reaction Dynamics, Dalian Institute of Chemical Physics, Chinese Academy of Sciences, Dalian 116023, China; 4 College of Chemistry and Chemical Engineering, Graduate School of the Chinese Academy of Sciences, Beijing 100049, China; E-Mails: luofang09b@mails.gucas.ac.cn (F.L.); jmj@gucas.ac.cn (M.J.); 5 Institute for Computational Science and Engineering, Laboratory of New Fibrous Materials and Modern Textile, The Growing Base for State Key Laboratory, Qingdao University, Qingdao 266071, China; E-Mail: sihz@qdu.edu.cn; 6 Department of Chemistry, Lanzhou University, Lanzhou 730000, China

**Keywords:** 3D-QSAR, molecular dynamics, MK-2 inhibitors, CoMFA, CoMSIA

## Abstract

Mitogen-activated protein kinase-activated protein kinase 2 (MK-2) has been identified as a drug target for the treatment of inflammatory diseases. Currently, a series of thiourea analogs as potent MK-2 inhibitors were studied using comprehensive computational methods by 3D-QSAR, molecular docking and molecular dynamics simulations for a further improvement in activities. The optimal 3D models exhibit high statistical significance of the results, especially for the CoMFA results with *r*^2^_ncv_, *q*^2^ values of 0.974, 0.536 for the internal validation, and *r*^2^_pred_, *r*^2^_m_ values of 0.910, 0.723 for the external validation and Roy’s index, respectively. In addition, more rigorous validation criteria suggested by Tropsha were also employed to check the built models. Graphic representation of the results, as contoured 3D coefficient plots, also provides a clue to the reasonable modification of molecules: (i) The substituent with a bulky size and electron-rich group at the C5 position of the pyrazine ring is required to enhance the potency; (ii) The H-bond acceptor group in the C3 position of the pyrazine ring is likely to be helpful to increase MK-2 inhibition; (iii) The small and electropositive substituent as a hydrogen bond donor of the C2 position in the oxazolone ring is favored; In addition, several important amino acid residues were also identified as playing an important role in MK-2 inhibition. The agreement between 3D-QSAR, molecular docking and molecular dynamics simulations also proves the rationality of the developed models. These results, we hope, may be helpful in designing novel and potential MK-2 inhibitors.

## 1. Introduction

Inhibition of the action of the proinflammatory cytokines TNF-α (Tumor necrosis factor-α) has illustrated both in animal models and in the clinic to be efficacious in the control of autoimmune diseases such as rheumatoid arthritis (RA) [1,2]. Numerous potential biological targets have been identified as being capable of inhibiting the TNF-α production, of which some have been used to design effective drugs for the treatment of inflammatory diseases. Among these targets, the most notable one is the kinase p38 MAPK, whose inhibitors demonstrate certain efficacy in both the preclinical *in vivo* models as well as the clinical trials in RA patients [3,4]. However, the first compound of this class has still not reached the market [5]. The lack of progress seems due to two main findings. On the one hand, drug toxicity observed for p38 inhibitors tested in the clinic may result in dose-limiting efficacy. On the other hand, p38 inhibition leads to the activation of multiple signaling pathways (such as ERK, JNK and so on) via a feedback control of TAK1 [6].

Mitogen-activated protein kinase-activated protein kinase 2 (MK-2), a direct substrate of p38, has been shown to play a central role in TNF-α production in mice genetically deficient in MK-2 [7,8]. It has been illustrated that MK-2 knockout mice show a strong reduction in disease incidence and disease severity scores in the arthritic CIA (collagen induced arthritis) model, and this reduction was also observed for the MK-2 heterozygote mice [9]. Moreover, MK-2 knockout mice are healthy and have a normal phenotype, while a genetic knockout of the p38 gene is embryonic lethal, suggesting an improved safety profile for MK-2 inhibition relative to p38 [10]. All these evidences suggest that a selective MK-2 inhibitor may exhibit an efficacy equal to that of a p38 inhibitor but without affecting additional cellular pathways governed by p38 that may lead to undesirable adverse effects [11]. Thus, inhibition of MK-2 provides a novel yet effective treatment for TNF-α-mediated diseases with little risk of side effects. Recently, several structural classes of compounds have been synthesized as MK-2 inhibitors, including the aminocyanopyridines [12], carboline analogs [10,13], tricyclic indole derivatives [8,14], benzothiophenes [15,16], thiourea analogs [3], spiro-3-piperidyl analogs [17], pyrrolopyridinone derivatives [18] and so on. Though these MK-2 inhibitors bear a certain amount of inhibitory activities, it is still difficult for these agents to obtain desirable characteristics to overcome inflammatory diseases. As such, developing the potential and selective MK-2 inhibitors is still a point of concern.

*In silico* modeling approaches [19–23], as a productive and cost-effective technology in the design of novel lead compounds, have been widely used in combination with experimental practices to facilitate the drug discovery process. Nevertheless, such computational studies on MK-2 inhibitors are still limited, with reports of only a single comparative molecular field analysis (CoMFA) research and pharmacophore modeling on pyrrolopyridine analogs [24,25], and a three-dimensional quantitative structural activity relationships (3D-QSAR) and docking modeling on carboline derivatives [26]. Yang and co-workers found that 3-, 4-positions of the phenyl ring could introduce bulky substituents and electronegative groups, respectively, which leads to the increase in potency; and bulky and electropositive groups at the 3-position of the quinoline are not favorable in these pyrrolopyridine analogs [24]. Investigations from Nayana *et al*. revealed that two hydrogen bond acceptors, one hydrogen bond donor, and one hydrophobic feature are essential for ligand binding [26]. Recently, the *in silico* model based on the pyrrolopyridine derivatives, reported by Kaushik and co-workers [25], identified the similar pharmacophoric features with that from Nayana: one hydrogen bond acceptor, two hydrogen bond donors, one hydrophobic group and one aromatic ring. But several questions remain unanswered: do other classes of MK-2 inhibitors also follow these rules? And if not, what are the possible rules for other molecules? To address this issue, in the present work, a more diverse set of thiourea derivatives, reported by Lin *et al*. [3] with less molecular weight and high potency *in vitro* activity against the MK-2 enzyme, were used to perform the computational study. In addition, besides 3D-QSAR methods, molecular docking and molecular dynamics (MD) were also performed to investigate the possible interaction mode between the potential thiourea derivatives and MK-2. Thus, in the present work, a comprehensive computational method combining 3D-QSAR, molecular docking and MD technologies was used to investigate a series of thiourea inhibitors of MK-2 in order to build predictive models and probe the possible interaction mode between these ligands and the target. The reliability and robustness of the developed best models were estimated by the bootstrapping analysis, 10 fold cross-validation and *y*-randomization test. In the current study, we are presenting, for the first time, a computational study on the series of thiourea compounds. The developed models could provide some helpful clues in the future synthesis of highly potent MK-2 inhibitors.

## 2. Results and Discussion

### 2.1. Selection of Training and Test Sets

**Figure 1:**
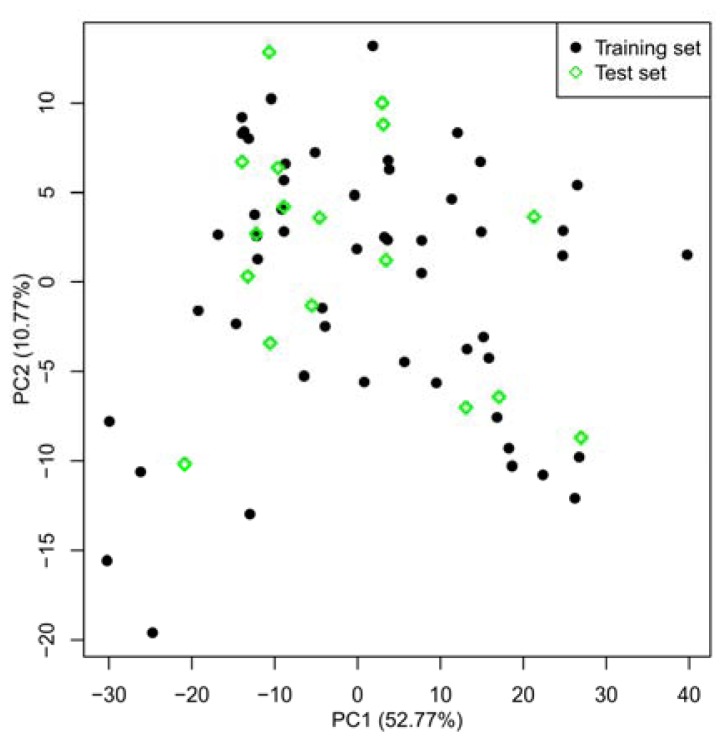
Principal component analysis of the Mold^2^ descriptors for MK-2 inhibitors.

The division of experimental datasets into training and test sets plays a crucial role in establishing a reliable QSAR model. Principal component analysis (PCA) can be employed for data survey which has been successfully applied to dataset split [27]. In the current study, in order to probe the descriptor space, a total of 777 molecular descriptors were calculated using the Mold^2^ software [28] for this series of inhibitors. After deleting zero- and near zero-variance descriptors, the remaining 493 descriptors were further studied. Figure 1 demonstrates the distribution of the compounds in the first two principal components, where the black dot denotes the training set, while green diamond stands for the test set. As seen from this Figure, on the one hand, representative points of the test set are close to those of the training set. On the other hand, the training and test sets uniformly fill the whole chemical space, indicating a rational division of the training and test data in the current work. The training set was applied for the development of the model and the external test set was used for the evaluation of predictive power of QSAR models. Finally, the training set and test sets include 53 and 18 compounds, respectively.

### 2.2. Results of CoMFA and CoMSIA

Of the various CoMFA and CoMSIA models generated in the present investigation, only the one with statistically significant results is selected for further analysis of the importance of each field individually. Table 1 shows both CoMFA and CoMSIA results obtained from the three different alignments (*i.e.*, I, from the database alignment; II, from docking alignment; III, from database alignment based on the docking conformations) using the training set compounds.

The CoMFA model from alignment-I yields the best statistical performances among all developed models, which presents the cross-validation coefficient *q*^2^ values of 0.536, standard error of prediction (*SEP*) of 0.642, with the principal components (*PCs*) of 8. The final non-cross-validation coefficient *r*^2^_ncv_ gives 0.974 and a standard error of estimation (*SEE*) of 0.151. The Steric and Electrostatic contributions (denoted by S and E) exhibit 49.6% and 50.4%, respectively.

As to the CoMSIA statistics obtained from the database alignment, a model with *q*^2^ = 0.556, *SEP* = 0.595, *PCs* = 3, *r*^2^_ncv_ = 0.779, *SEE* = 0.420, *F* = 57.509 was obtained. Three field discriptors of S, E, Donor (D) present 0.250, 0.503 and 0.247, respectively.

As can be seen from Table 1, the models from the alignments II and III cannot obtain statistically significant results in terms of internal and external predictive performances. Thus, our main analysis is restricted to the alignment I models for the prediction of MK-2 inhibitors.

For the optimal CoMFA and CoMSIA models, besides the leave-one-out (LOO) validation, the cross-validation in groups using 10 folds repeating 10 times was also carried out, where the mean value of *r*^2^_cv(mean)_ was reported. To further assess the robustness and statistical confidence of the derived models, a bootstrapping analysis for 100 runs was also performed, and we gave the average *r*^2^ and *SEE* values of these 100 runs (namely, *r*^2^_bs_ and *SEE*_bs_). Table 1 illustrates all corresponding results, where the *r*^2^_bs_ and *SEE*_bs_ values are 0.989 and 0.099 for the optimal CoMFA model as well as 0.820 and 0.374 for the best CoMSIA model based on the alignment I, respectively, indicating the robustness of the present built models. In addition, the *r*^2^_cv(mean)_ values from the whole dataset for both the CoMFA and CoMSIA models are 0.630 and 0.606, proving the highly statistical significance of current models.

The *y*-randomization validation was also performed to the best CoMFA and CoMSIA models. Herein, we randomized the target values of 50 times and rebuilt the models. As a result, none of those models had significant *q*^2^. The obtained *q*^2^ values of the check are in the range from −0.612 to 0.101 for the best CoMFA and from −0.392 to 0.107 for the optimal CoMSIA. All these operations indicate that the obtained *q*^2^ values for both the best CoMFA and CoMSIA models with original data are not due to chance correlations, elucidating the fact that both the models possess a real prediction power for the current dataset.

**Figure 2:**
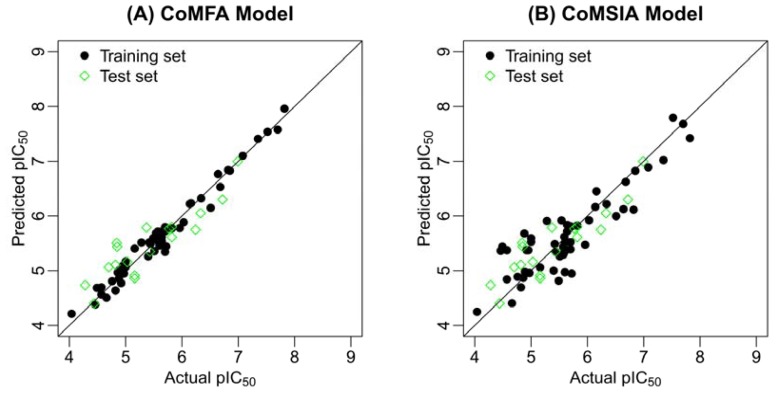
The predicted *versus* the actual pIC_50_ values for the MK-2 inhibitors. (**A**) CoMFA model and (**B**) CoMSIA model.

**Figure 3:**
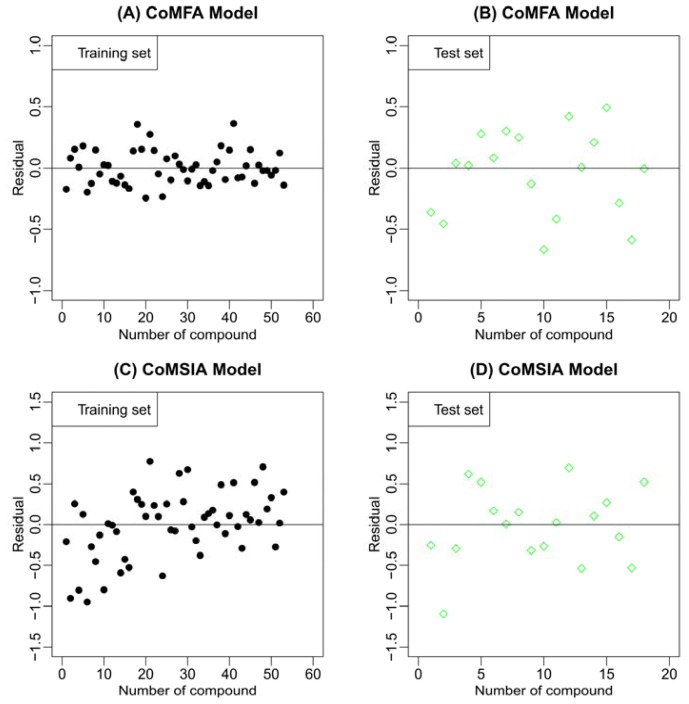
The residual plots for the optimal CoMFA and CoMSIA models. CoMFA residual plots for the training and test sets are shown in (**A**) and (**B**), respectively; CoMSIA residual plots for the training and test sets are shown in (**C**) and (**D**), respectively.

It is well-known that external validation is the only way to establish a reliable QSAR model. Here, the root-mean squared error of prediction (RMSEP) for the test set is reported, where RMSEP values of CoMFA and CoMSIA are 0.342, 0.451, respectively. According to references [29–31], models are considered acceptable if they satisfy all following conditions: (1) *r*^2^_pred_ > 0.5; (2) *r*^2^_test_ > 0.6, where *r*^2^_test_ is conventional coefficient for the test set; (3) (*r*^2^_test_ − *r*^2^_o_)/*r*^2^_test_ < 0.1, where *r*^2^_o_ is the coefficient without intercept for the test set data; (4) 0.85 ≤ *k* ≤ 1.15, where *k* is the slope when the predicted values of the test set compounds (*X* axis) are plotted against the observed values of compounds (*Y* axis) with the intercept set to zero; (5) *r*^2^_m_ > 0.5, where $r_{m}^{2}=r_{test}^{2}\times \sqrt{r_{test}^{2}-r_{o}^{2}}$. Table 2 shows all the check results of the best CoMFA and CoMSIA models for the external test set. It is clear that both the CoMFA and CoMSIA models satisfy the rigorous criteria, where the former gives the better results. Table 3 gives the experimental and predicted pIC_50_ values for the best CoMFA and CoMSIA model. Figure 2 illustrates the correlation plots of the experimental *versus* the predicted pIC_50_ values of the training (black dot) and test (green diamond) sets for the two best 3D-QSAR models. Figure 3 gives the residual plots for optimal CoMFA and CoMSIA models. Clearly, good correlations are observed since the predicted values are almost as accurate as the experimental activities for the whole dataset (especially for the CoMFA model, see Figures 2,3). Thus, based on the calculated statistical parameters, one can come to the conclusion that the present models can accurately predict the activity of novel compounds of this series, suggesting that the developed 3D-QSAR models are reasonable.

### 2.3. Contour Maps

**Figure 4:**
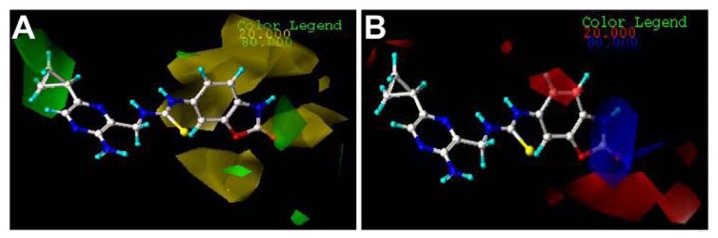
CoMFA StDev*Coeff contour plots with the combination of compound 70.

**Figure 5:**
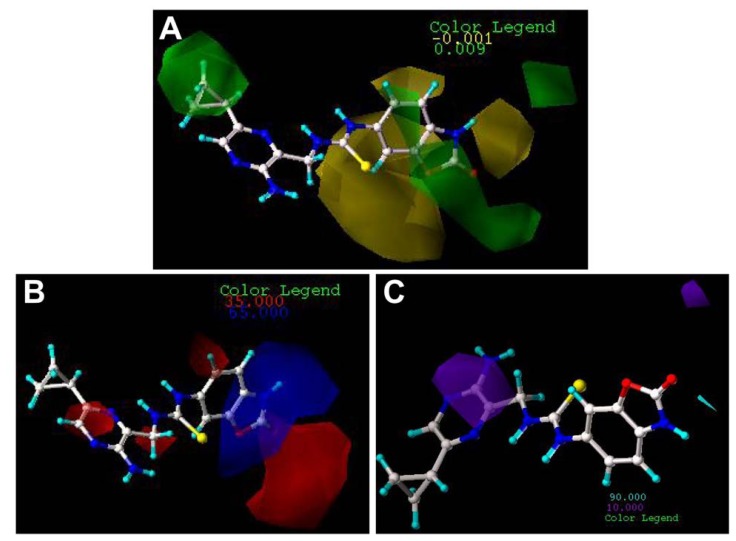
CoMSIA StDev*Coeff contour plots with the combination of compound 70.

The CoMFA and CoMSIA results are represented as 3D coefficient contour maps which show regions where variations of different fields in the structural features of the molecules lead to the increase or decrease of the activity. In this study, the most potent inhibitor 70 is exhibited as a representative molecule in the following CoMFA and CoMSIA contour maps (Figures 4,5).

The steric and electrostatic fields from the best CoMFA model are represented in Figure 4. In the steric field (Figure 4A), the green-colored contours represent the regions of favorable steric effect, while yellow-colored contours represent regions of unfavorable steric effect.

As shown in Figure 4A, there exists a large green contour surrounding the *c*-Pr of compound 70, indicating that the presence of a bulky group in this position will induce an increase of the inhibition activity for the class of compounds. The observation is fully supported by the experimental results. Compounds 1, 13, 14, 15 and 16, having a corresponding substituent instead of hydrogen in position 5, respectively, exhibit higher activities compared to inhibitor 12. This suggests that an introduction of substituent in this position 5 is necessary for the high potency. That is the same case for compounds 66 and 67. The other green-colored maps appear at the distal of the carbonyl group in the oxazolone, it is also observed that there are yellow-colored maps around the benzo-oxazole, suggesting that a careful selection of substituent is necessary. The exploration is in agreement with the experimental results. A system comparison of compounds 1–11 can provide a clue of how to select a proper group to enhance the activity. For inhibitors 2 and 3, the corresponding substituents (Et and *i*-Pr) point to the yellow-colored contour maps, which lead to the decreased activities. However, the counterpart 4 with the *t*-Bu gives about 10-fold higher potency compared with compounds 2 and 3, since one of the branches of *t*-Bu is near to the green-colored map. Generally speaking, it can be found by inspecting the contour maps that bulky groups near the oxazole positions are not tolerant for MK-2 inhibition (compounds 6–10), because these groups are enmeshed in the bulky-forbidden zones. The reason why compound 11 presents relatively high activity is that the 1-Naphthyl group is closer to the green-colored maps instead of the yellow contour maps. For the series of phenyl-substituted compounds 17–43, one can notice that the green-colored maps are located in the 2- and 4-positions, while the yellow-colored ones are in the 3-position. All these investigations can be supported by Lin’s results that mono-substitution on the 4-position or the 2-position of the phenyl group is clearly preferred over the 3-postion, with 3–10 fold differences in potency. Similarly, 2,4-disubstitution exhibits better activity compared with 3,5-disubstitution [3]. Since there are several yellow-colored maps which cover the benzo-oxazole group, the larger substituent will damage the MK-2 inhibitory activity in the positions. An investigation of compounds 44–60 comes to the conclusion that substituting hydrogen in the –NH group with –Ph (compound 49) or –Me (compound 54) will result in significant loss of potency, since the branch substituents of these compounds are embedded in the yellow-colored maps.

The CoMFA electrostatic contour map with the most potent compound 70 is shown in Figure 4B. Blue contour maps mean that positive-charged substituent groups are beneficial for the activity, while red contours indicate that negative charges are conducive. As shown in Figure 4B, two little red contour maps are observed surrounding the *c*-Pr of the pyrazine ring, suggesting that a charge-rich group near these positions enhances biological activity. Compared with compound 13 with a methyl group in the position, compound 1 with the electron-rich chlorine group exhibits 2-fold higher activity. That is the same case for compounds 48 and 52. It can be seen that there is one red-colored region in front of the S atom, which may be why all these series compounds have the electron-rich group in this position. The other red contour maps are near the oxazolone group, suggesting that in the positions it is beneficial to activity to the introduction of electron-rich groups. It can be seen that compounds 52 and 53 give the higher activity, since the R_1_ groups with the negative group are surrounded by red contour maps. It is the same case for the compounds 68–70. In addition, a large blue-colored region exists around the –NH group of the oxazolone, illustrating that the positive group is favored to enhance the inhibitory activity. It is clear that compounds 44–48, compounds 52 and 53 and compounds 66–71 exhibit the high activities, since the –NH groups of these compounds are all embedded in the blue-colored maps.

Since the CoMSIA steric and electrostatic contour maps (Figure 5A,B) are similar to the CoMFA model, they are not discussed here further. Figure 5C depicts the H-bond donor contour map of the CoMSIA model. Cyan contours encompass areas where an H-bond donor leads to improved biological activity, while a donor located near the purple regions results in the loss of biological activity. Clearly, it is easily found that a cyan-colored map surrounds the –NH group of the oxazolone, indicating the presence of an H-bond donor group in this region for the activity. The investigation is also supported by previous CoMFA and CoMSIA electrostatic contour maps where in these positions a large blue map exists, indicating electron-withdrawing groups, as hydrogen bond donors are beneficial to increase the potency of these inhibitors. The investigation is also consistent with the previous report [3] where the free –NH is found to be necessary to keep the high potency for the carbamate series (compounds 44–60), as substituting it with a phenyl group (compound 49) or a methyl group (compound 54), or replacing it with oxygen (compound 55) resulted in significant loss of potency. In addition, a purple-colored contour map is near nitrogen of the pyrazine ring, indicating the disfavor regions for H-bond donor groups. In fact, in this position, hydrogen bond acceptor groups are favored as depicted in the docking results which will be discussed in the following part. It is also noted that a small purple map is near to the carbonyl group of the oxazolone. This suggests that a hydrogen bond acceptor in the position is beneficial to the MK-2 inhibitory activity. Thus, this information obtained from the CoMFA and CoMSIA contour maps is helpful to understand the interactions between the inhibitors and MK-2.

### 2.4. Molecular Docking

**Figure 6:**
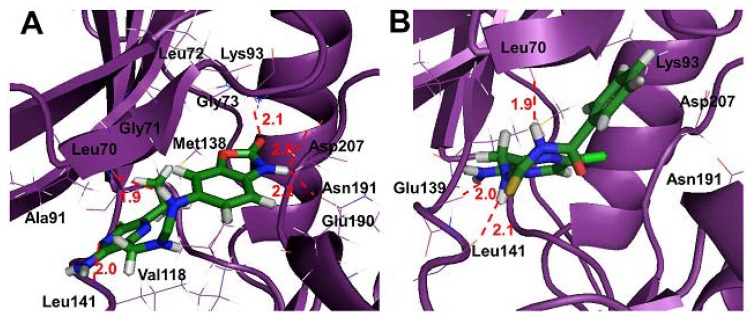
The binding result of compounds with MK-2. The ligands are colored in forest green and key amino acid residues in black labels. H-bonds are shown in red dash lines. (**A**) Binding result of the highest active ligand 70 with MK-2; (**B**) Binding results of the lowest active ligand 10 with MK-2.

As we know, the docking results could illustrate us the interaction models between inhibitors and MK-2 (PDB entry code: 3KGA). In the present work, the most potent molecule 70 and the lowest active compound 10 are taken as the representative compounds to elucidate this point in detail. As illustrated in Figure 6A, the compound 70 is located at the binding site by interaction with amino acid residues of the receptor through a hydrogen bond and van der Waals interactions. The compound 70 is represented as a carbon-chain in forest green and key residues in MK-2 as a carbon-chain in brown line mode. It can be noted in this Figure that the *c*-Pr group of the inhibitor is sandwiched between the glycine loop and the hinge region of MK-2, with good lipophilic interactions with the side chain of Leu70 of the glycine loop and the main chain of Met138, Val118 and Ala91, which accommodate a hydrophobic substituent in this position. The observation is in agreement with the previous report [11]. In addition, the docking results can be supported by our CoMFA and CoMSIA steric maps where the large group here will increase the inhibitory activity. The pyrazine nitrogen of the compound is within a reasonable distance (2.0 Å) of forming a hydrogen bond with the main chain nitrogen of Leu141. Velcicky *et al*. also proved the importance of Leu141 in the ligand binding [32]. The H-bond donor contour map of CoMSIA also suggests that an acceptor group in this position is favored. In addition, one of the nitrogen atoms of thiourea forms a hydrogen bond with the main chain oxygen of Leu70 (1.9 Å), which is in agreement with the previous report [26]. This hydrogen bond may be the particular characteristic compared with pyrrolopyridines, aminopyrazoles and benzothiophenes, due to the flexible thiourea chain. More importantly, the oxazolone moiety forms three hydrogen bonds in the binding region of MK-2. The oxygen of oxazolone forms one hydrogen bond to the terminal nitrogen of the conserved lysine (Lys93, 2.1 Å). The oxazolone nitrogen engages the side chain carboxylate of the conserved aspartic acid (Asp207, 2.8 Å) and forms another hydrogen bond with the side chain oxygen of Asn191 with the hydrogen bond length of 2.2 Å. All these interactions fix the ligand in a desirable direction. The investigations are fully supported by CoMFA and CoMSIA electrostatic and H-bond donor contour maps. In the literature [15,16,32–34], parts of the interactions are also observed. The aryl ring, which is important for potency, primarily makes van der Waals contacts with the residues of Glu190, Gly71, Leu72 and Gly73. Lovering *et al*. also come to the conclusion that the glycine-rich loop plays a central van der Waals role to accommodate the hydrophobic moiety in the ligand [33]. In Figure 6B, it can be noted that the –NH_2_ group attached to the pyrazine ring forms one hydrogen bond with Glu139, and two nitrogen atoms in the flexible thiourea chain also yield two hydrogen bonds with Leu141 and Leu70, respectively. However, there are some important hydrogen bond interactions missing in this compound, with the important amino acid residues, such as Lys93, Asp207, which may be the reason why this ligand presents low inhibitory activity.

### 2.5. MD Simulations

**Figure 7:**
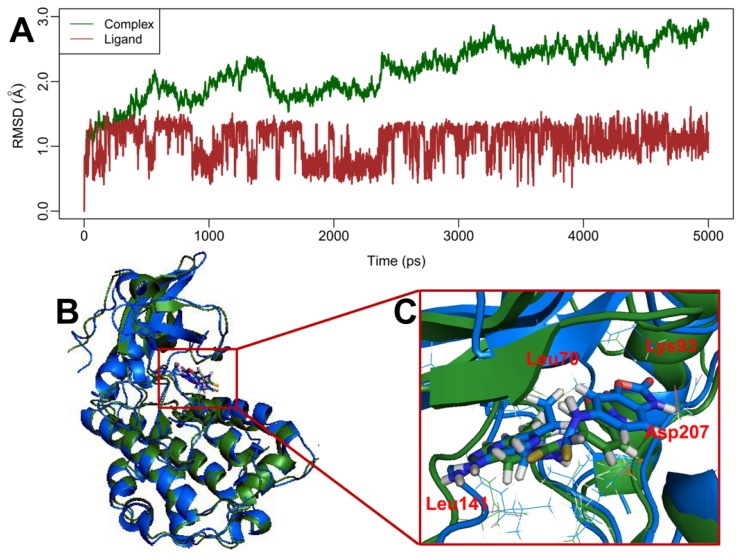
(**A**) Plot of the root-mean-square deviation of docked complex/ligand *versus* the MD simulation time in the MD-simulated structures; (**B**) Distant view of superimposed backbone atoms of the time-averaged structure from the final 2 ns of MD simulations (colored in forest green) and the initial structure (colored in blue) for compound 70 with MK-2; (**C**) Nearby view the alignment at the active center of MK-2.

In the current investigation, a 5 ns molecular dynamics simulation of the docked complex of MK-2 with inhibitor 70 was performed to obtain a dynamic picture of the conformational changes that occur in an aqueous solution, with the main emphasis to investigate the conformational change that takes place in the inhibitor and the enzyme (Figure 7).

Figure 7A shows root mean square deviation (RMSD) (calculated by the backbone atoms) of the trajectory for the complex with respect to the initial structure (colored in dark green). The Figure shows that a relatively stable conformation of the complex is achieved after 3 ns simulations with RMSD value of 2.5 Å. Figure 7A also gives the RMSD (calculated by atoms apart from hydrogen atoms) of the ligand 70 (colored in brown) in the MK-2 binding site. It can be noted that the RMSD for the ligand reaches about 1.0 Å in the beginning of MD simulations and retains this value throughout the process, indicating the reliability of ligand during molecular dynamics simulations.

In order to validate the reliability of the docking results, we also compared the results between the MD and docking simulations in terms of H-bond interactions and van der Waals contacts. By and large, the interaction modes produced between MD and docking simulations share common features. The van der Waals interactions formed in the docking study can also be found in the MD simulations. The common amino acid residues for both simulations are Ala91, Leu70, Met138 and val118 which interact with a hydrophobic *c*-Pr substituent, Glu190, Gly71 and Lue72, forming van der Waals contacts with the aryl ring. In addition, it can be noticed that there are three common H-bond interactions between MD and docking researches. The residues of Lys93, Asp207 and Leu141 can form H-bonds with the ligand investigated by both simulations. Furthermore, it can also be noticed that there exists slight differences among docking and MD simulations. For the MD results, it can be found that there are three H-bonds between ligand and target, while the docking results form six H-bonds. After a careful exploration, the possible reason of the decreased number of H-bonds in MD simulations is that the MK-2 target used in MD is derived from homology modeling, which causes the target to be slightly different from the crystal one. Thus, the flexible loop frame is easy to move while performing MD simulations.

In order to compare the structures from MD simulations and docking, a superimposition of both the structures is shown in Figure 7B,C, where the blue cartoon represents the docked structure, while the forest green one stands for the time-averaged structure from the final 2 ns of MD simulations, with compound 70 represented as a stick, colored the same with the corresponding enzyme, respectively. It can be noticed that from this Figure, there is basically no significant difference between the structures from MD simulations and docking. Although the loop of MK-2 has undergone slight movements during MD simulations, conformations of binding pocket and ligand are still stable, suggesting the rationality and validity of the docking model.

Since the previous reports have also been performed based on the pyrrolopyridine and carboline analogs as MK-2 inhibitors [24–26], it is very interesting to compare both similarities and differences between our studies and theirs. One the one hand, all these investigations yield the satisfied prediction power in terms of *q*^2^ and *r*^2^_pred_. In addition, some common structural features of MK-2 inhibitors have been identified: there exists one hydrogen bond acceptor and one hydrogen bond donor at least in these compounds. The common key amino acid residues have been found to play a central role in inhibitory activity of MK-2 in our and Nayana’s investigation, such as Leu141, Met138, Leu70, Asp207 and Lys93. On the other hand, the present study also exhibits several different points as follows: (1) The present studied system is on the basis on the novel thiourea derivatives with low molecular weight and high potential for the inhibition of MK-2; (2) besides the conventional 3D-QSAR and molecular docking researches, the recently popular MD simulations are also used to investigate this series of thiourea analogs; (3) the more rigorous validating criteria suggested by Tropsha and Roy as well as *y*-randomization are also adopted to check the developed models, and importantly the optimal models completely pass all these tests. In summary, all the findings obtained by the previous reports and ours complement each other and offer a clue to designing novel MK-2 inhibitors.

## 3. Materials and Experimental Methods

### 3.1. Dataset

A series of 71 MK-2 inhibitors were collected from the literature [3] after removing one outlier (11a, depicted in literature [3]). Here, the converted molar pIC_50_ (−logIC_50_) values were used as the dependent variables in the QSAR regression analysis to improve the normal distribution of the experimental data points. The whole data set was divided into training (53 molecules) and test (18 molecules) sets, respectively considering the structural diversity and the uniformly distribution of activity values. The structures and the corresponding activities are depicted in Table 3.

### 3.2. Molecular Modeling and Alignment

The 3D structures of all compounds were constructed by using the sketch molecule modules of the SYBYL6.9 package. Partial atomic charges were calculated by the Gasteiger-Hückel method, and energy minimizations were performed by using the Tripos force field and the Powell conjugate gradient algorithm with a convergence criterion of 0.005 kcal/mol.

**Figure 8:**
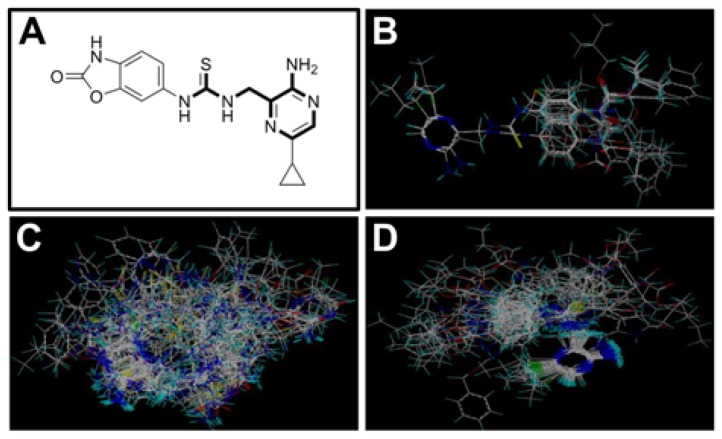
The alignment of all molecules in the dataset. (**A**) Inhibitor 70 is used as the template for molecular alignment, with the bold part as the common scaffold; (**B**) Alignment I: from the database alignment; (**C**) Alignment II: from the direct molecular docking conformations; (**D**) Alignment III: from the combination of both alignments I and II.

Molecular alignment of compounds is an important step for the successful development of 3D-QSAR models. Thus, in the present work, three alignment methods were used to build the models. Alignment I: In this process, each derivative was aligned to the template by rotation and translation so as to minimize the RMSD between atoms in the template and the corresponding atom in the analog using the “DATABASE ALIGN” option in SYBYL [35,36]. For the current work, the most potent compound 70 was chosen as a template to fit the remaining training and test sets of compounds. Thereafter, all compounds finally minimized with the lowest energy in the dataset were aligned to a common substructure shown in the bold part in Figure 8A by a substructure-based alignment method using the align database technology implemented in SYBYL (Figure 8B). Alignment II: the alignment from the direct molecular docking conformations (Figure 8C). Alignment III: the combination of both alignments I and II, which means that the active conformation of each compound with the highest score was directly extracted from molecular docking, and then all these molecules were aligned together using the database alignment method as that of alignment I. Figure 8D presents this resulting alignment result.

### 3.3. Docking Simulation

Docking simulations of thiourea analogs into the MK-2 binding pocket were performed using the Surflex-dock method. For the current study, the X-ray crystal structure of MK-2 was retrieved from RCSB Protein Data Bank (PDB ID: 3KGA) with resolution of 2.55 Å. Prior to docking, the ligand and other sub-structures were extracted from the crystal structure, and hydrogen atoms were added to the protein in standard geometry using the biopolymer modulators. In this study, Auto-based Mode was adopted to generate the protomol in Surflex-dock program. Two adjustable parameters that affect the size and extent of the generated protomol are the threshold and the bloat values which were set to 0.5 and 0, respectively. Other parameters were adopted by default values in the Surflex-dock. In the current work, the maximum number of poses per ligand was set to 10. The conformations with the highest total scores for ligands of the data set were aligned automatically together inside the binding pocket of MK-2.

### 3.4. CoMFA and CoMSIA Calculations

The steric and electrostatic field energies were calculated using a *sp*^3^ probe atom with a charge of +1.0 at all intersections of a regularly spaced grid of 2.0 Å in all three dimensions with the defined region. The van der Waals and Coulomb-type potentials representing the steric and electrostatic fields, respectively, were calculated using the Tripos force fields. The grid box dimensions were determined by the automatically created features in the CoMFA module. Both the steric and electrostatic energy values were both truncated to 30.0 kcal/mol.

In CoMSIA, the steric, electrostatic, hydrophobic, hydrogen bond donor and hydrogen bond acceptor potential fields were also calculated at each lattice intersection of a regularly spaced grid of 2.0 Å as that used in CoMFA. A probe atom with radius 1.0 Å and a charge of +1.0 with hydrophobicity of +1.0 and hydrogen bond donor and acceptor properties of +1.0 were used to estimate the steric, electrostatic, hydrophobic, donor and acceptor fields. The attenuation factor *α* was set to 0.3.

### 3.5. Statistical Validation

In the current study, the CoMFA and CoMSIA descriptors served as independent variables and the pIC_50_ values as dependent variables in PLS analysis for the development of 3D-QSAR models. The predictive values of the models were evaluated first by the leave-one-out cross-validation process. The cross-validated coefficient, *q*^2^, was calculated using Equation 1:

(1) $$q^{2}=1-\frac{\sum_{i=1}^{train}{\left(y_{i}-{\hat{y}}_{i}\right)}^{2}}{\sum_{i=1}^{train}{\left(y_{i}-{\bar{y}}_{tr}\right)}^{2}}$$

where *y_i_*, *ŷ_i_*, and *ȳ_tr_* are the observed, predicted, and mean values of the pIC_50_ values, respectively for the training set. Herein, the term $\sum_{i=1}^{train}{\left(y_{i}-{\hat{y}}_{i}\right)}^{2}$, is the predictive residual sum of squares (*PRESS*).

The optimal number of components obtained from the cross-validation was used to derive the final QSAR model. Then, a non-cross-validation analysis was carried out; and the Pearson coefficient (*r*^2^_ncv_), *SEE* and the *F* values were calculated. Finally, the CoMFA and CoMSIA results were graphically represented by field contour maps, where the coefficients were generated using the field type “Stdev*Coeff”.

As has been reported [29], the low value of *q*^2^ for the training set can exhibit a low predictive ability of a model. However, the opposite is not necessarily true. That is, the high *q*^2^ is necessary, but not sufficient, for a model with the high predictive power. Therefore, the external validation must be estimated to establish a reliable and predictive QSAR model. The predictive correlation coefficient *r*^2^_pred_ listed in the following equation was used to validate the models. In addition, various criteria suggested by Tropsha and Roy [29,31] were also performed to estimate the predictive power of the current built models

(2) $$r_{pred}^{2}=1-("PRESS"/SD)$$

where *SD* is the sum of the squared deviations between the actual activity of the compounds in the test set and the mean activity in the training set, and “*PRESS*” is the sum of the squared deviations between predicted and observed activity for each compound in the test set.

### 3.6. Molecular Dynamics Simulations

Due to the fact that the incomplete crystal structure of MK-2 is obtained (PDB: 3KGA, resolution: 2.55 Å), this crystal structure from the Protein Data Bank was used as the template to make the complete model of MK-2. The structure of the MK-2 model was generated by homology modeling using SWISS-MODEL [37]. The MK-2 sequence was obtained from the UniProt database (Accession number: P49137) [38].

To identify a functionally validated complex from protein docking and the most potent molecule 70, we performed 5 ns molecular dynamics simulations to investigate the conformational changes in the complex induced by the ligand 70. The software AMBER 11 [39] was used for the MD simulations. The inhibitor was minimized using the HF/6-31G* optimization in Gaussian03, and the atom partial charges were obtained by fitting the electrostatic potentials, Gaussian derived, via the RESP fitting technique in AMBER 11. The force field parameters for the molecules were assigned by the Antechamber program [40] in AMBER 11. Herein, the AMBER FF03 [41] and GAFF [42] force fields were used for protein and ligand, respectively. Hydrogen atoms were added to the protein and all of the crystal water molecules were retained with *tleap* module from AMBER. The system was then put in to a rectangular box of TIP3P water molecules [43].

The whole systems were minimized in three stages to remove bad contacts between the complex and the solvent molecules. First, the water molecules were minimized with the atoms of protein restrained; Second, water and the side chains of the protein were minimized by restraining the backbone of the protein, and each stage was performed by using the steepest descent minimization of 2500 steps followed by a conjugate gradient minimization of 2500 steps. Third, the minimization of the entire system was performed without restraint by 10,000 steps, changing the minimization method from steepest descent to a conjugate gradient after 5000 cycles. After 15,000 steps minimization and equilibration for 60 ps, the system was then heated gradually from 0 to 310 K in the NVT ensemble and equilibrated at 310 K for another 60 ps. After minimization and heating, 5 ns MD simulations were performed at a constant temperature of 310 K and a constant pressure of 1 atm. The SHAKE algorithm [44] was applied to fix all bonds involving hydrogen atoms throughout the MD simulations, and the long-range electrostatics were treated with the Particle-Mesh Ewald (PME) method [45].

## 4. Conclusions

Presently, a class of thiourea analogs as potent MK-2 inhibitors for curing inflammatory diseases has been investigated for the purpose of developing 3D-QSAR models derived from both ligand- and receptor-based superimpositions. Statistically significant models have been obtained from two 3D-QSAR methods of CoMFA and CoMSIA on the basis of the database alignment. The CoMFA model presents a higher predictive power than CoMSIA expressed in terms of several rigorous evaluation criteria, such as *q*^2^, *r*^2^_pred_, *k* and *r*^2^_m_, for both the internal and external data sets. In addition, both methods have successfully passed through the *y*-randomization test, suggesting the robustness of the built models. Graphical interpretation of the optimal results, provided by the CoMFA and CoMSIA analyses, presents the crucial structural features that could be responsible for the activity of MK-2 inhibitors illustrated as follows: (i) The substituent with a bulky size and electron-rich group at the C5 position of the pyrazine ring is required to enhance the potency; (ii) The H-bond acceptor group in the C3 position of the pyrazine ring is likely to be helpful to increase the MK-2 inhibition; (iii) The small and electropositive substituent as a hydrogen bond donor of the C2 position in the oxazolone ring is favored; (iv) The key amino acid residues (*i.e.*, Leu141, Lys93, Asp207) have been found to be important to form the hydrogen bond interactions with the series of thiourea derivatives.

In sum, in this report, several reliable computation models, including 3D-QSAR, molecular docking and molecular dynamics have been used to investigate a class of thiourea analogues as potential MK-2 inhibitors. The optimum models not only exhibit good statistical performances, but also provide a better understanding of interaction modes between the ligand and the target at the molecular level. The developed models could provide some instructions for further synthesis of highly potent MK-2 inhibitors in a drug discovery process.

## Figures and Tables

**Table 1:** The CoMFA and CoMSIA results based on different alignment methods.

PLS analysis	Alignment methods					

	I		II		III	
		
	CoMFA	CoMSIA	CoMFA	CoMSIA	CoMFA	CoMSIA
*q*^2^	0.536	0.556	−0.035	0.263	0.311	0.400
*SEP*	0.642	0.595	0.909	0.759	0.741	0.699
*PCs*	8	3	3	2	3	4
*r*^2^_ncv_	0.974	0.779	0.873	0.810	0.897	0.843
*SEE*	0.151	0.420	0.318	0.385	0.287	0.357
*F* value	207.641	57.509	112.475	106.868	141.747	64.605
*r*^2^_cv(mean)_	0.630	0.606	0.169	0.273	0.401	0.390
*r*^2^_bs_	0.989	0.820	-	-	-	-
*SEE*_bs_	0.099	0.374	-	-	-	-
*r*^2^_pred_	0.810	0.669	0.416	0.480	0.444	0.156

**Relative Contribution (%)**						

S	0.496	0.250	0.348	-	0.475	1.000
E	0.504	0.503	0.652	0.483	0.525	-
H	-	-	-	0.517	-	-
D	-	0.247	-	-	-	-
A	-	-	-	-	-	

**Table 2:** Results of the optimal CoMFA and CoMSIA models for the external prediction set.

Model	*r*^2^_pred_	*r*^2^_test_	(*r*^2^_test_ − *r*^2^_o_)/*r*^2^_test_	*k*	*r*^2^_m_
CoMFA	0.810	0.807	0.013	0.993	0.723
CoMSIA	0.669	0.677	0.054	0.998	0.548

**Table 3:** The structures and experimental and predicted activities (pIC_50_ in M) of the best CoMFA and CoMSIA models for MK-2 inhibitors.

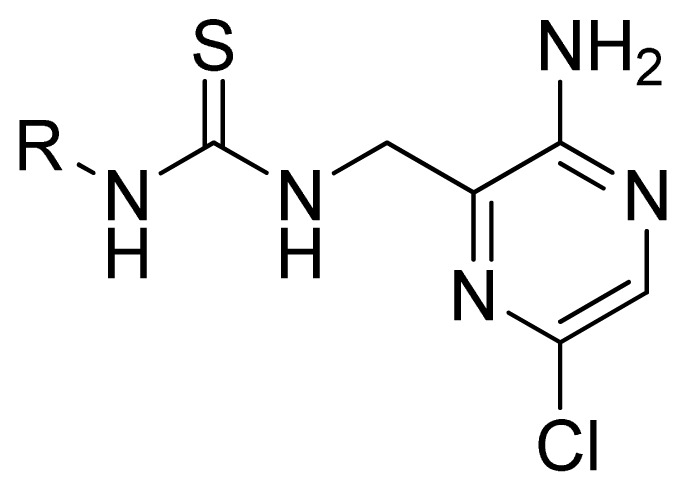					

Compd.	R		Experimental pIC_50_	Predicted pIC_50_	

				CoMFA	CoMSIA
1	Ph		5.699	5.344	5.391
2*	Et		4.699	5.063	4.954
3	*i*-Pr		4.886	5.013	4.978
4	*t*-Bu		5.602	5.569	4.973
5	*c*-Pr		4.921	4.773	5.374
6*	*c*-Pentyl		4.276	4.735	5.375
7	*c*-Heptanyl		4.456	4.380	5.364
8*	Bn		4.444	4.401	4.736
9	MeOCH_2_CH_2_		4.658	4.507	4.405
10	Benzoyl		4.041	4.213	4.249
11	1-Naphthyl		5.495	5.595	4.816

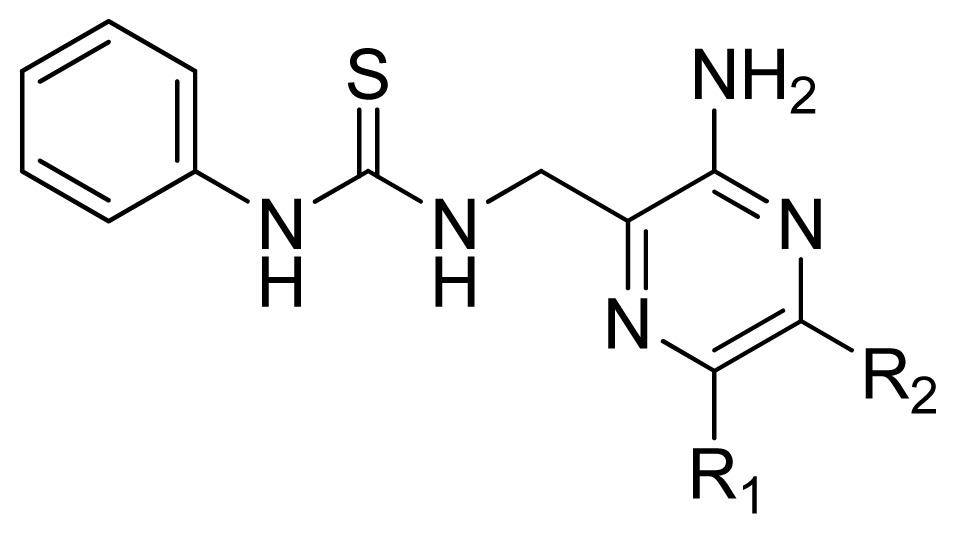					

**Compd.**	**R_1_**	**R_2_**	**Exprimental pIC_50_**	**Predicted pIC_50_**	

				**CoMFA**	**CoMSIA**

12	H	H	4.495	4.687	5.440
13	Me	H	5.398	5.261	5.001
14 *	Et	H	5.824	5.799	5.203
15	*n*-Pr	H	5.638	5.614	5.837
16 *	*c*-Pr	H	6.328	6.051	5.811

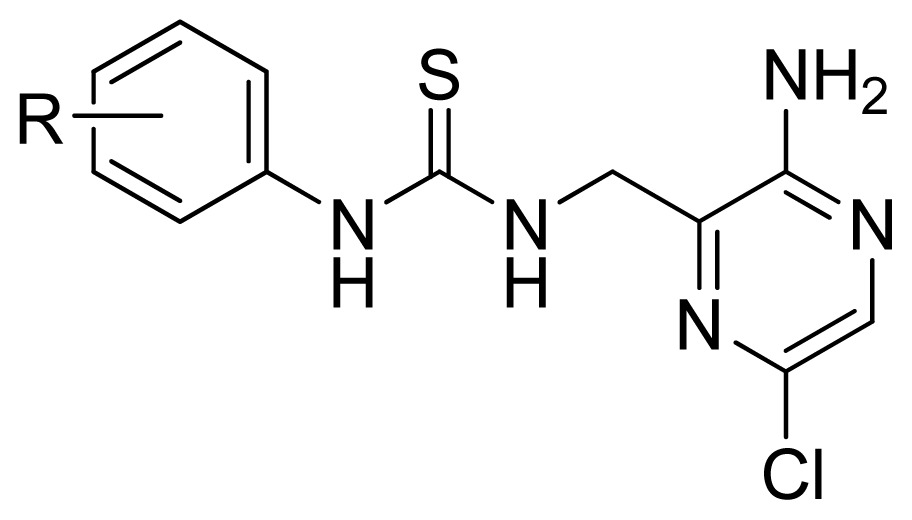					

**Compd.**	**R**		**Experimental pIC_50_**	**Predicted pIC_50_**	

				**CoMFA**	**CoMSIA**

17	4-Me		5.569	5.714	5.436
18 *	4-Cl		5.432	5.348	5.263
19	4-MeO		5.721	5.444	4.948
20	4-*i*-Pr		5.585	5.516	5.337
21	4-*t*-Bu		4.863	4.968	4.868
22	4-Br		5.509	5.357	5.261
23	4-NO_2_		5.004	5.168	5.527
24 *	4-CN		5.161	4.859	5.153
25	4-BnO		5.569	5.582	5.290
26 *	4-Ac		5.161	4.910	5.010
27	4-EtOC(=O)-		4.971	4.949	4.958
28	4-NMe_2_		5.155	5.405	5.060
29	4-(Morphorin-1-yl)		4.762	4.809	4.889
30	4-AcNH-		5.284	5.515	5.908
31	4-NH_2_		5.699	5.795	5.811
32	4-BnOC(=O)NH-		6.337	6.322	6.218
33	2-Me		5.444	5.488	5.342
34	2-Cl		5.959	5.778	5.473
35	2-MeO		5.585	5.599	5.619
36	2-F		5.699	5.720	5.524

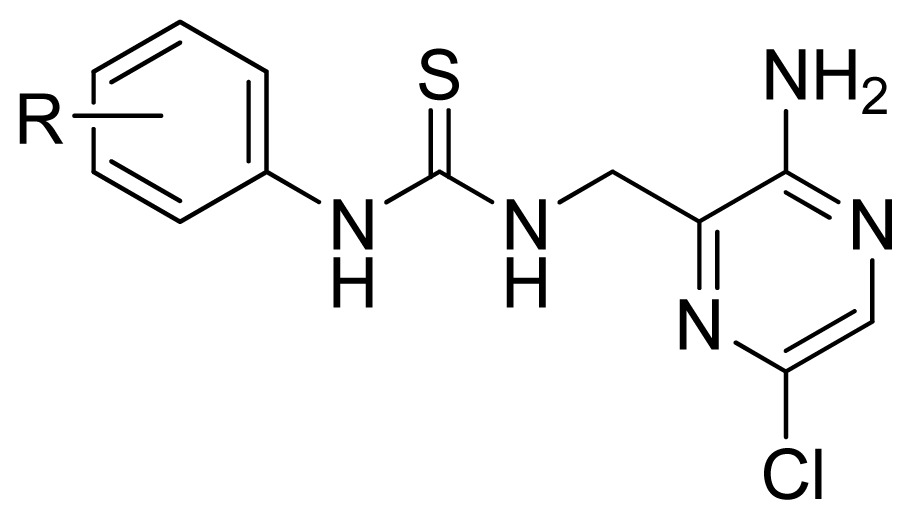					

**Compd.**	**R**		**Experimental pIC_50_**	**Predicted pIC_50_**	

				**CoMFA**	**CoMSIA**

37	2-MeS		5.602	5.711	5.511
38 *	3-Me		5.032	5.160	5.348
39	3-Cl		4.947	5.088	5.375
40 *	3-MeO		4.839	5.507	5.107
41	2,4-Di-MeO		5.602	5.457	5.365
42 *	2,4-Di-Cl		5.367	5.787	5.345
43	3,5-Di-Cl		4.569	4.564	5.374

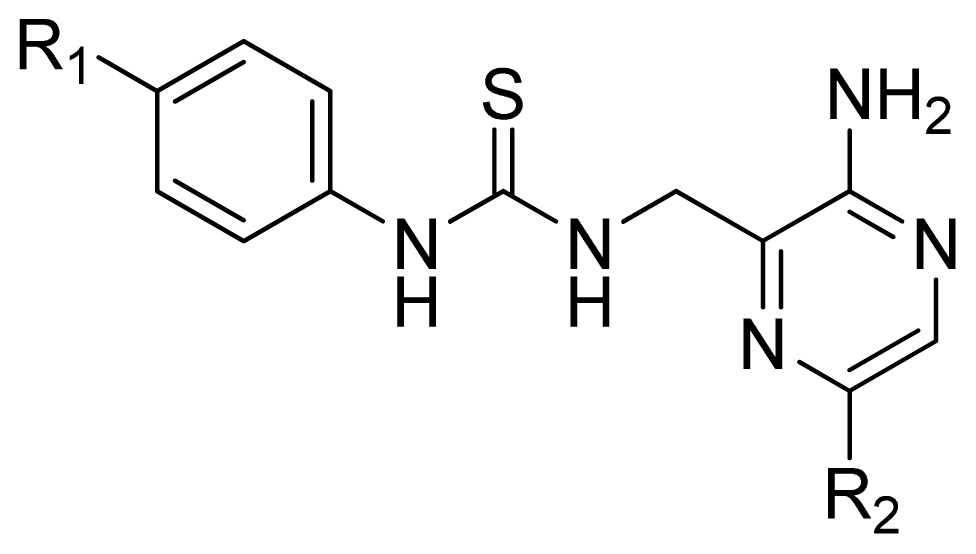					

**Compd.**	**R_1_**	**R_2_**	**Experimental pIC_50_**	**Predicted pIC_50_**	

				**CoMFA**	**CoMSIA**

44	MeOC(=O)NH-	Cl	6.027	5.884	5.920
45	EtOC(=O)NH-	Cl	6.509	6.147	5.997
46	*n*-BuOC(=O)NH-	Cl	6.638	6.765	6.124
47	*i*-BuOC(=O)NH-	Cl	6.824	6.841	6.114
48 *	*t*-BuOC(=O)NH-	Cl	6.721	6.299	6.025
49	*i*-BuOC(=O)N(Ph)-	Cl	5.824	5.772	5.824
50 *	MeNHC(=O)NH-	Cl	5.770	5.765	6.309
51	*t*-BuNHC(=O)NH-	Cl	6.155	6.234	6.451
52	*t*-BuOC(=O)NH-	Me	6.678	6.530	6.624
53	PhOC(=O)NH-	Me	6.137	6.221	6.165
54	EtOC(=O)N(Me)-	Me	5.000	5.067	5.592
55	*t*-BuOC(=O)O-	Me	5.538	5.684	5.918
56	EtC(=O)NH-	Me	4.876	4.854	5.679
57	*n*-PrC(=O)NH-	Me	5.638	5.541	5.719
58 *	*n*-BuC(=O)NH-	Me	5.824	5.612	5.715
59	BnC(=O)NH-	Me	5.420	5.518	5.487
60 *	PhC(=O)NH-	Me	6.237	5.748	5.971

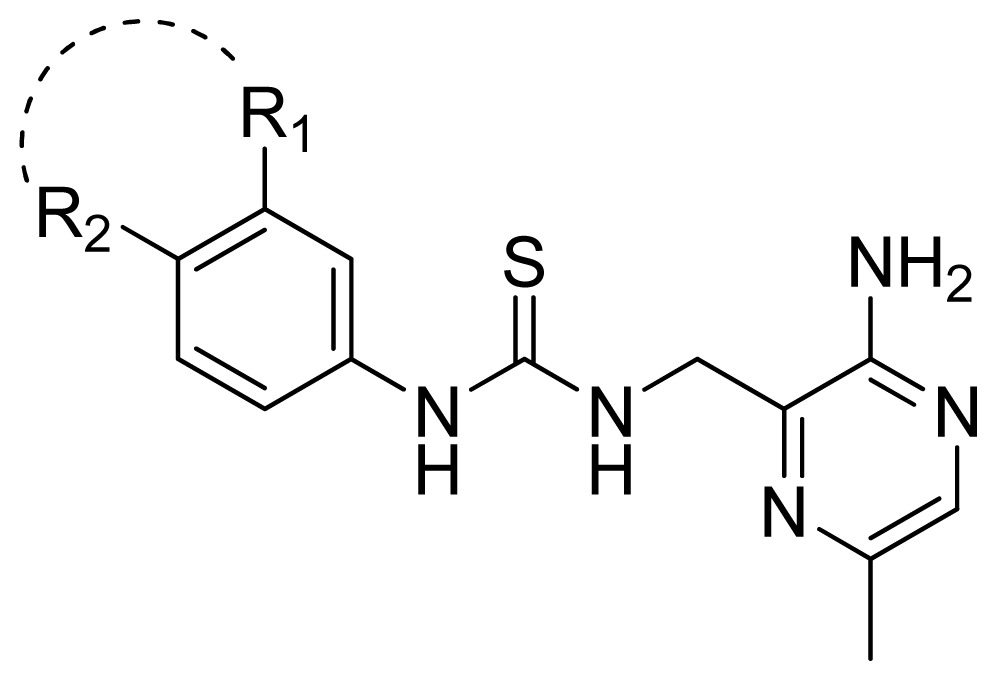					

**Compd.**	**R_1_-R_2_**		Experimental pIC_50_	**Predicted pIC_50_**	

				**CoMFA**	**CoMSIA**

61	3-NHC(=O)NH-4		4.824	4.639	4.696
62	3-CH_2_C(=O)NH-4		4.569	4.696	4.842
63 *	3-SC(=O)NH-4		4.824	5.106	4.972
64 *	3-NHC(=O)O-4		4.854	5.437	5.382

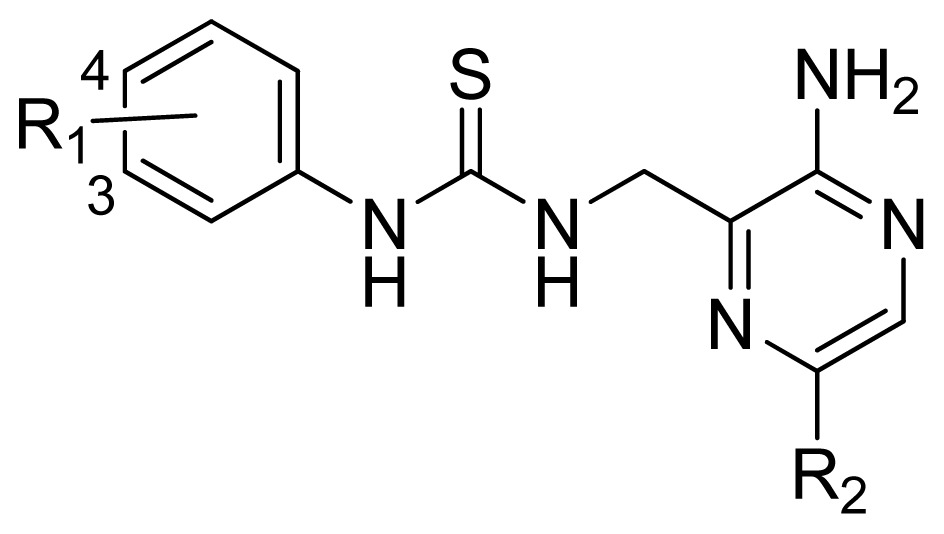					

**Compd.**	**R_1_**	**R_2_**	**Experimental pIC_50_**	**Predicted pIC_50_**	

				**CoMFA**	**CoMSIA**

65	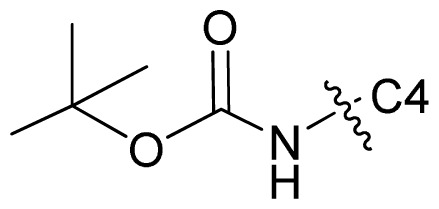	Et	6.854	6.827	6.824
66	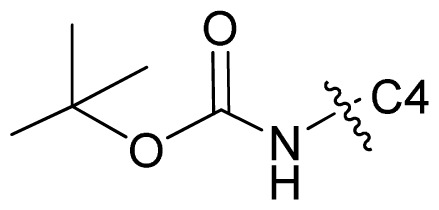	*n*-Pr	7.347	7.409	7.020
67*	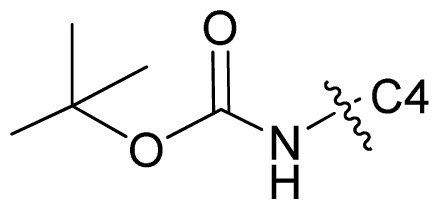	*c*-Pr	6.987	6.996	6.470
68	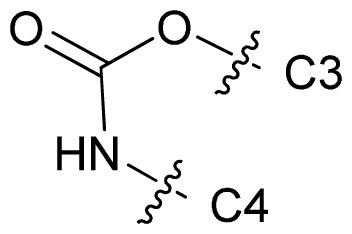	*n*-Pr	7.699	7.577	7.681
69	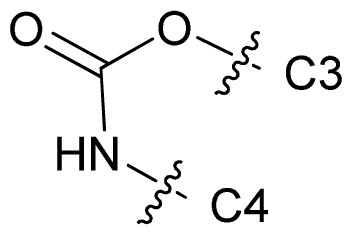	*n*-Bu	7.523	7.539	7.795
70	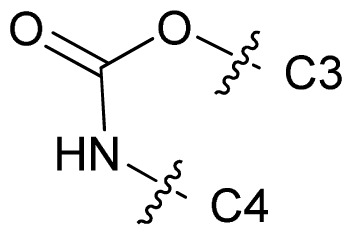	*c*-Pr	7.824	7.960	7.422
71	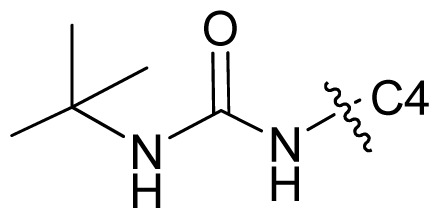	*c*-Pr	7.081	7.100	6.889

* denote the test set.
